# Cytoprotective mechanism of cerebro-cognitive reserve

**DOI:** 10.1192/j.eurpsy.2024.1333

**Published:** 2024-08-27

**Authors:** A. Sidenkova

**Affiliations:** Psychiatry, Ural State Medical University, Yekaterinburg, Russian Federation

## Abstract

**Introduction:**

Consideration of the reserve problem would be incomplete without an analysis of the cytoprotective mechanism. The predominant molecular hallmark of aging and degeneration is the accumulation of altered gene products. Moreover, several conditions, including protein, lipid, or glucose oxidation, disrupt redox homeostasis and lead to the accumulation of unfolded or misfolded proteins in the aging brain in case of AD, and other neurodegenerative diseases that have as a common denominator abnormal protein production, mitochondrial dysfunction and oxidative stress. Some authors classify aging, pathological aging, and neurodegeneration as “protein conformational diseases”.

**Objectives:**

scientific publications

**Methods:**

analytical review

**Results:**

The central nervous system has evolved a conserved unfolded protein response mechanism to cope with the accumulation of misfolded proteins. As one of the main intracellular redox systems involved in neuroprotection, the vitagene system becomes a potential neurohormetic target for novel cytoprotective interventions. Vitagens encode the cytoprotective heat shock proteins (Hsp) Hsp70 and heme oxygenase-1, as well as thioredoxin reductase and sirtuins. The cellular stress response is the ability of a cell to withstand stressful conditions, including the heat shock response. The production of heat shock proteins, including protein chaperones, is necessary for the folding and repair of damaged proteins, which promotes cell survival to avoid apoptosis.«Molecular chaperone» are proteins that function as part of an ancient defense system in our cells. They promote cell survival by sequestering damaged proteins and preventing their aggregation. Chaperone complexes are involved in the regulation of mitochondrial functions, assembly of the cytoplasmic proteolytic system of brain cells. The cellular response to stress requires the activation of survival pathways that are under the control of protective genes called vitagens. Vitagens are involved in the production of heat-shock protein molecules, glutathione, and bilirubin. They have antioxidant and anti-apoptotic activity and provide protection against oxidative stress.

**Conclusions:**

Studies have shown that the heat shock response contributes to the maintenance of cellular homeostasis, the establishment of a cytoprotective state in a wide range of human diseases, including inflammation, cancer, aging, and neurodegenerative disorders. Endogenous proteins can be manipulated by food or pharmacological compounds, which represents an innovative approach to therapeutic intervention in neurodegenerative disorders, actually influencing reserve mechanisms and adaptive capacity.

**Disclosure of Interest:**

None Declared

